# Cancer of the Throat: A Physician’s Experience as a Patient

**DOI:** 10.5041/RMMJ.10252

**Published:** 2016-07-28

**Authors:** Itzhak Brook

**Affiliations:** Professor of Pediatrics, Georgetown University School of Medicine, Washington DC, USA

**Keywords:** Cancer, depression, laryngectomy, medical errors, throat

## Abstract

The author, a practicing physician, was diagnosed with throat cancer and lost his vocal cords. He endured the side effects of radiation, repeated surgeries, and the effects of prolonged hospitalizations; confronted medical mistakes and discrimination after losing his vocal cords; and struggled to regain his speech and find new meaning and purpose for his life. Facing the hardship and trials of becoming a laryngectomee illustrated to him how dependent and helpless a patient can become. Being unable to speak, eat, and breathe normally, while dealing with a potentially terminal illness, makes the patient very vulnerable, both physically and emotionally. A skillful, competent, error-free, empathetic, and caring approach that recognizes what the patient is experiencing can expedite recovery and well-being and help the patient return to a productive and meaningful life.

## INRODUCTION

This manuscript describes my personal experiences enduring several surgical procedures, including radical neck dissection and laryngectomy for removal of pyriform sinus squamous cell carcinoma. As an infectious diseases clinician with an interest in head and neck infections, I had little knowledge or experience in the management of head and neck cancer. I was suddenly thrust into an unfamiliar and challenging reality where I had to cope with a life-threatening malignancy as a patient rather than in the role of a clinician. In addition to experiencing the untoward effects of radiation treatments, surgery, and prolonged hospitalizations, I suffered from diagnostic and therapeutic errors related to my care, as well as discrimination. It was a struggle to learn to speak again and a challenge to find new purpose for my life.

I share these experiences with the hope that medical providers will better recognize and appreciate the hardships faced by their patients diagnosed with cancer. My goal is to encourage proficient, competent, error-free, and compassionate care, and to facilitate patient recovery and well-being.

## FACING THROAT CANCER

I was shaken to the core upon being diagnosed with hypo-pharyngeal cancer. I refused to believe my diagnosis until I had seen the pathological specimens with my own eyes.^1^ I had to accept facts that I had previously avoided—I was vulnerable and my life might be shortened due to this illness. I had always believed I would stay in good health and be free of any serious diseases. As I grew older I began to accept the inevitable reality that illness might ultimately lead to my demise; however, such thoughts were for a far distant future. Without warning I faced an unforeseen danger and the potential of imminent death. Although my cancer had been detected and removed at an early stage (T1, N0, M0) and had not spread, my prognosis was unclear. I was left with a lingering fear that the cancer would return or metastasize.

Recovering from the side effects of radiotherapy (altered taste, inflamed mucosa, reduced saliva production, hypothyroidism) was a lengthy process. As time passed I began to believe I might be cancer-free. However, a year and a half after completing radiotherapy my symptoms reoccurred—pain and difficulty swallowing. Although I hoped otherwise, a biopsy revealed the return of the malignancy.

I did not share my diagnosis with many others at first—only family and work colleagues. I did not want to appear weak or helpless or be stigmatized and discriminated against. However, with the cancer’s return I could no longer hide my illness—future surgeries might lead to my disfigurement. I was surprised to find that as I began sharing my diagnosis with others, the feelings of isolation and embarrassment passed and I greatly benefited from their much-needed support and sympathy.

## SELECTING THE APPROPRIATE TREATMENT

With the recurrence of the cancer, two treatment options were presented to me: removal of my entire larynx versus removal of only a portion of my larynx. After further consideration my physicians proposed preserving my vocal cords by using only laser surgery to remove the cancerous tissue. However, the final decision was up to me. I therefore consulted with several head and neck cancer specialists from the US and abroad. I asked each one about the advantages and disadvantages of the different surgical approaches for my specific cancer (T2, N0, M0). Some believed in performing total laryngectomy while others believed that laser, when used by experienced operators, would work. Wishing to save my vocal cords and to avoid major surgery, I finally chose laser surgery.

I now had to select the hospital where the surgery would be performed. One hospital was out of town; although they had a great deal of experience with trans-oral laser microsurgery for head and neck cancer, there was a long waiting time. The other hospital was close to home and could schedule the procedure much earlier. It was the hospital where I practiced, and I knew the physicians personally. However, they had minimal experience with this surgery. In considering my options, the earlier surgical date, familiarity with the staff, and the fact that I felt too emotionally and physically drained to travel were the deciding factors: I selected the local hospital.

To my great disappointment, despite three separate attempts within three weeks, the surgeons were unable to remove the tumor. Initially they had been optimistic. However, after each procedure the final pathological report returned showing residual cancer. Over those three weeks my life turned into a draining emotional up-and-down ride from initial feelings of elation to disappointment and despair.

Having failed to remove the cancer, my surgeons began planning a partial or complete laryngectomy. However, I was beginning to doubt their competence and sought the opinion of a surgeon who was more experienced in treating my kind of cancer; he practiced at a large medical center in a different city.

My new surgeon recommended radical surgery that evolved into a total laryngectomy. He explained that this surgery offered the highest chances of survival and ultimate cure for someone in my condition. I agreed. I had to wait an additional four weeks, but the surgery was successful, and the malignancy was excised in its entirety. To date the cancer has not returned locally or systemically.

## HOSPITALIZATION FOLLOWING THE SURGERIES

I had no way of knowing what I would feel or endure following the operations. I experienced sedation, frailty, dizziness, and clouded sensations. Being unable to eat or drink by mouth and relying on tube feedings only grew more difficult over time. I began to have dreams where I was eating and drinking normally again, and was terrified to be caught since it was forbidden to me. I lay helpless in bed, linked up to catheters, suction and oxygen tubes, intravenous and arterial lines, and connected to monitoring equipment. Blood samples were taken multiple times a day, and an intravenous line had to be reinserted more than once. Over time, finding venous access became increasingly difficult. I had to be suctioned multiple times a day to remove the accumulated mucus and sputum. Swallowing saliva was so painful that I spit it out or let it drool down my face. Unable to speak, the only way I could communicate was by writing on a chalkboard or in a notebook.

Adjusting to this new living reality was more than difficult. At this point in my treatment, I began to understand why some individuals elected to forgo life-extending interventions. This realization was new to me—I had always supported prolonging patients’ lives. For the first time it dawned on me that prolonging life might not always be the optimal choice.

The constant presence of family members and friends became increasingly difficult and taxing. Yes, even though they were supportive, and ensured that I received appropriate care, sometimes I wanted to be left alone, longed for privacy, and just wanted to rest. Dealing with my visitors’ needs for reassurance and support was also difficult. They meant well, but at times their extended stays and conversations were an additional burden to me.

## PHYSICIANS’ ATTITUDE

I encountered two different kinds of physicians throughout my fight with cancer: optimists and pessimists.

Being a medical professional made it easier for me to navigate the medical system, obtain treatment, and get answers to questions from the majority of my doctors. I preferred clinicians who were willing to discuss all the possible risks, complications, and my long-term prognosis, even if it was not a good one. I was aware of their professional constraints and was grateful whenever they were open, sincere, and accepted responsibility for their errors. Several of my doctors probably concluded that I understood and knew more about my illness and treatment options than I did. I repeatedly had to ask them to explain everything to me and not to assume that I knew everything. Colleagues with whom I had previously worked gave me greater individual attention than those with whom I had not.

Being a clinician myself, I always wanted the full details about my condition including my test results and differential diagnoses. Expressing my opinions on familiar issues was at times helpful. I also tried to keep my physicians apprised regarding any new symptoms and signs that might help in their care for me. Doing this helped them recognize that my cancer had returned.

However, after losing the ability to speak, being only able to write made communication much more difficult. Some of my surgeons had no patience to “listen” in this manner. They always seemed to be in a rush to continue their medical rounds and get back to the operating room or clinic. They spent as little time as possible by my bedside, often two to three minutes, and only examined my upper airways and surgical site. Most did not even carry a stethoscope, and I had to insist that they listen to my chest. Many of the nurses were inconsistent in their examinations and often ignored important physical systems.

Frustrated by my surgeons’ superficial approach and unable to get the input I needed from them, I began writing down all my inquiries and concerns before the medical rounds. This ensured that I got some responses. However, I rarely had an opportunity to ask new or follow-up questions during the rounds. All too often they left me with numerous unanswered inquiries and I felt upset and ignored.

Thankfully, some surgeons and residents were more attentive and showed greater sensitivity and compassion. However, I also observed how the impatient, insensitive, and rushed attitude of some senior surgeons served as an inappropriate role model to many physicians in training.

On several occasions I actually faced very rude clinicians. Once, my tracheotomy tube became obstructed and I urgently needed it cleaned. I asked a senior resident to perform the task. He did so grudgingly, rinsing it out with tap water instead of the proper sterile cleaning kit and water. As he finished, I could still feel that the tracheotomy tube was clogged. However, he refused to clean it again with the appropriate cleaning kit. He harshly uttered: “We call the shots here,” and exited the room. This humiliating experience again left me feeling helpless and quite upset. When I told the attending surgeon about the incident he seemed uninterested took no action to reprimand the resident.

Despite these upsetting events, I am deeply grateful to the clinicians, nurses, and technicians who cared for me. The removal of my larynx and the reconstruction of my upper airways were impeccable. The majority of the professionals who cared for me were empathetic and personable and conveyed their compassion and sympathy.

## THE POWER OF A HUG

In some situations, the best thing your medical professional can do is to give you a hug. I first experienced this after I informed my internist that my throat biopsy revealed the presence of cancer. Without saying a word, my physician approached me and gave me a warm hug. I was distraught after learning about the cancer’s recurrence, and his hug conveyed that he felt my pain and distress. I realized that I would not be alone in fighting the cancer and that I would be supported by medical professionals who sincerely cared for me. The hug was a genuine act of sustenance and sympathy and was given at the moment I needed it most.

This spontaneous gesture of human contact was a refreshing return to the fundamentals of medicine that has been forgotten in modern medicine; all too often technology and tests replace human contact. A “hug” can be manifested in a variety of ways. It can be expressed through a pat on the back, a heartfelt handshake, or by eye contact. Indeed, there is scientific confirmation that human contact can lead to the production of chemicals such as oxytocin and endorphins that can reduce pain and generate calmness and happiness.^2^,^3^

## MEDICAL MISTAKES

The care I received from medical professionals at clinics and hospitals was generally very good. However, errors were made by physicians, nurses, medical technicians, and clerks, at all levels of my care. As a medical professional, I recognized many errors and was able to prevent some of them. Unfortunately, patients without a medical background may not be able to recognize and abort mistakes and are more likely to suffer their consequences.

Realizing how prevalent medical errors are was shocking to me. Although I have been practicing medicine for over 40 years, I had never observed so many mistakes in patient care as while I myself was a patient. This was probably because I had never before suffered from a serious illness nor been hospitalized for a serious illness. After becoming a laryngectomee I had difficulty preventing and reporting many of these errors. However, I rapidly learned that it was up to me to ensure that mistakes did not occur.

The first mistake occurred when my otolaryngologist attempted to remove my hypopharyngeal tumor via laser surgery, but instead excised scar tissue. The error was not recognized until a week later because the excised tissues were not inspected using frozen sections in the operating room. Two additional surgeries followed, but the tumor was not removed in its entirety. Post-surgical edema contributed to the difficulty in removing the entire malignancy during the repeat surgeries.

I was exposed to several hazardous situations because of nurses’ errors. While still in the surgical intensive care unit a mucous plug suddenly blocked my airways. I wanted to alert the nurses but my call button had fallen to the floor. I attempted to get the nurses’ attention by unplugging my monitors one by one. Although my room was in front of the nurses’ station, no one came to my aid despite the sounding of the alarm signals from all my monitors. No help came for at least 10 minutes, and then only because my wife entered my room and saw that I was having trouble breathing.

Multiple mistakes were made by the hospital staff, including failing to wash hands or use gloves when needed; taking oral temperature without covering the thermometer with protective plastic; taking blood pressure with cuffs that were too small or too large; administering incorrect medication doses or delivering drugs by mouth instead of through the nasogastric tube; improper suctioning of mucous and cleaning of the tracheal tube; failing to connect the call button; forgetting to enter physician orders to my chart, and entering a different patient’s orders to my chart.

The result of the last-mentioned error was that oral feeding was started prematurely, 7 days after my laryngectomy instead of 14 days later. I only suspected that this was inappropriate because my surgeon had previously informed me when oral feeding would be resumed. Despite my repeated request to get a confirmation from a senior surgeon, oral feeding was continued for almost 18 hours until the error was recognized. The error apparently occurred because an order to start oral feeding for another patient was entered to my chart.

Miraculously these incidents did not cause long-term harm. However, once I appreciated the prevalence and the magnitude of these errors, I started questioning every order and procedure made by the medical professionals. This made my hospital stay very unpleasant and stressful.

Conveying my concerns whenever I observed errors was very difficult because I was unable to speak, was very weak, and was receiving medications that blurred my sensations. I also suppressed my desire to question or criticize the medical staff because of my dependence on them; I did not want to anger or upset them. I realized that the only way to prevent mistakes was by fending for myself and to become my own advocate without fear of repercussions.

I tried to get the assistance of the hospital’s patient advocates several times but was disappointed at their reluctance to intervene or inability to help. I realized how important it is for a patient to have a family member or friend act as their advocate. I was fortunate that my family members were able to report some of the errors; this led to improvement in the care I received.

The way to prevent medical mistakes is for the medical team to talk honestly about them with their patients. Medical errors reduce patients’ trust in the system and the people who care for them. Admitting and accepting responsibility for a mistake, when appropriate, and having an open dialogue about it can restore trust and contribute to patients’ well-being. Once a patient realizes that his/her concerns are acknowledged and steps are taken to prevent future errors they can start relaxing and concentrate on their recovery.^4^

## LIFE AFTER LARYNGECTOMY

Even though my surgeons attempted to prepare me for total laryngectomy and its aftermath, it was difficult for me fully to absorb the information I received during the difficult days prior to surgery. Hence, I was not fully able to deal with life afterward. No words could convey the feelings and consequences of becoming a laryngectomee until I actually became one. It was impossible to appreciate the reality of my losses, which included losing my voice, all sensations in parts of my face and neck, and the ability to smell.

My daily life changed in unanticipated ways, and many things that I took for granted were gone. I had to contend with reflux of food and liquid when bending; the inability to swallow dry food or to converse during meal times; reduced neck and shoulder range of motion; no sensation over the skin donor site on my left arm; having a stoma in my neck, edema of my face and neck, and losing my Adam’s apple; repeated bouts of uncontrolled coughing; the constant need to clean my voice prosthesis and trachea; obtaining supplies needed to speak with a voice prosthesis and carrying them with me at all times; ensuring that my stoma was covered at all times with a base plate properly glued to my skin; learning to speak using a voice prosthesis and facing technical problems in its maintenance; losing my voice when the voice prosthesis was blocked; being misunderstood when speaking in noisy places or over the phone; difficulties in expressing feelings or laughing; and facing discrimination.

The above obstacles and unforeseen hardships made my life challenging—to say the least. I had to find a way to eat and speak again and find a meaningful and productive way of living. I constantly reminded myself that dealing with these difficulties was better than the alternative of dying from cancer.

## OVERCOMING THE FEAR OF RECURRENCE AND DEPRESSION

I faced depression, hopelessness, and fear of cancer recurrence following my surgeries. Initially, feeling depressed had enabled me to face being newly diagnosed with cancer and feeling hopeless had a paradoxical calming effect. Fortunately, I quickly realized that giving up on life would send the wrong message to my children and I would set the wrong example for them. However, after becoming a laryngectomee, battling depression became a constant fight when confronted with new norms and restrictions. My low levels of thyroid hormone, anemia, and physical exhaustion further contributed to the down feeling.

I learned that there were some things I could do to reverse the depression. Slowly returning to enjoyable and gratifying tasks such as teaching, caring for my own patients, and writing, were all very helpful. My family and most of my friends were supportive of my endeavors, and my colleagues and patients accepted me without reservation.

Also instrumental to overcoming the feelings of depression and hopelessness were a local laryngectomee support group, a psychologist, a caring and supportive head and neck surgeon, and a speech and language pathologist (SLP). I also took advantage of the advice and camaraderie available over the Internet. There are several laryngectomee and head and neck patient support group sites where head and neck cancer survivors from across the globe help each other with advice and support. This is a treasured resource for individuals traveling a similar road.

Fear of the cancer’s return was particularly acute before taking tests to monitor my condition, such as computed tomography and positron emission tomography scans. I slowly learned to accept living with the continuous uncertainty of a cancer diagnosis; as time passed my anxiety subsided.

## LEARNING TO SPEAK AGAIN

Relearning how to speak was an uphill struggle full of frustration and pitfalls. Speech was impossible for more than nine weeks following my laryngectomy. An electrical larynx would have helped me to speak sooner, but that option was not offered to me. The guidance of an experienced and dedicated SLP immediately after losing my vocal cords was most helpful. However, the SLP who worked with me after I was discharged from the hospital was not as knowledgeable and did not recognize my need for more guidance and follow-up. I quickly learned that the field of speech pathology is both an art and a science; each SLP has her or his unique approach to solving problems, and I could benefit from all of them.

Finding what worked best for me was a trial and error process.^5^ I had to learn how to cope with voice prosthesis leakage, find out the optimal method for placing and sealing the baseplate for the heat–moisture exchanging filter, and perfect the way I spoke.

My voice came out in a soft rusty whisper after my laryngectomy—it barely resembled my original voice. Speaking was a laborious task that involved straining the muscles of my rib cage and diaphragm to push air forcefully into the voice prosthesis. Unexpected coughing interfered with my speech, and if my prosthesis got obstructed by mucus, speech was impossible. Also challenging was the expression of emotion through my voice or altering vocal intensity.

As mentioned above, the help and guidance of a devoted SLP was instrumental in regaining and improving my speech. Teamwork between my SLP and my otolaryngologist was invaluable in finding solutions to many issues. Unfortunately, collaboration between SLPs and otolaryngologists does not always happen, as many ear nose and throat specialists have minimal familiarity with and expertise in the care of voice prostheses and speech restoration.

Mastering the ability to speak and especially to give a lecture was difficult. Unfortunately, I received no training on how to speak correctly following my laryngectomy. It was only six years later that a new SLP embarked on an intense four-month speech training program and taught me to speak properly and effortlessly. This was done with the assistance of an expiratory intra-tracheal air pressure manometer. I practiced how to speak slowly, producing minimal air pressure (almost a whisper), to over-articulating words, and learned to take a breath every four to five words. This was a difficult and slow process, since my manner of speaking before laryngectomy had been rapid and flowing. I learned to economize when speaking by delivering the essentials of each topic using fewer words. After mastering these techniques lecturing became much easier. In turn, my self-confidence increased and I no longer dreaded failing when giving lectures.

## EXPRESSING MYSELF IN WRITING

The difficulties in expressing myself through speaking drove me to convey my ideas in writing. I decided to share my personal journey by writing a book^6^ that describes what happened to me in the three years following my cancer diagnosis. I shared how I faced and dealt with various treatments and how I adjusted to the new reality of life after surgery. I described my worries, doubts, concerns, fears, frustrations, disappointments, and ultimate acceptance of my handicaps, and the adjustment to living with the permanent question as to what will come next. I wrote motivated by the hope that medical professionals would better understand the difficulties and uphill battle a patient with cancer faces, and the critical role they can play in providing their patients with the best care possible.

I also created a website^7^ and wrote a guidebook^8^ offering useful medical, dental, and psychological information to assist head and neck patients, including laryngectomees. Therein I discuss the side effects of radiation treatment and chemotherapy; speaking methods post-laryngectomy; airway management; stoma and voice prosthesis care; eating and swallowing issues; medical, dental, and psychological challenges; respiratory emergency care; pain relief; and help with traveling.

Writing these books helped me turn a trying and challenging period of my life into something helpful for others. I chose to change this lemon into lemonade!

## PERSONAL REFLECTION AND OUTCOME

I know that I underwent the optimal procedure to remove the primary tumor although it meant losing my voice and suffering the lifelong side effects of radiation therapy. I was, and remain, grateful for the additional years I gained through my physicians’ care, and the many joyful new moments experienced such as helping other people, seeing my grandchildren grow, and traveling to new places.

Resumption of my old life occurred one step at a time: I began reading professional literature again; I had to learn to shower without getting water in my lungs; I began walking outside in my neighborhood; eventually I resumed bicycling and climbing mountains. One of the most gratifying moments came when I was able to deliver a lecture again. Even though I was very dependent on the microphone because of my weak voice, it was an important milestone.

I had to accept the reality that I could not deliver a lecture as I had before, nor speak as clearly and forcefully as presenters with intact vocal cords. However, I have learnt that even with my poor voice I can still address important issues that can impact patient care and generate greater compassion for patients with serious illnesses. Through lecturing I found new meaning and purpose to my life that helps myself as well as others. I am invigorated by turning the loss of my vocal cords into a means for doing good. By sharing my personal experiences with patients and health professionals, others can benefit. In this arena, my imperfect voice has become an advantage rather than a handicap—it sends a powerful message to the audience.

Experiencing serious illness as a patient has significantly influenced how I relate to my patients. It has made me more caring, empathetic, and sensitive. I better appreciate what patients and their families endure. I do my best to help them know they are not alone, to provide them with more attention and support, and to be sensitive and understanding of the magnitude of their loss. I strive to follow the example of the health care providers who helped me the most. My personal experiences have taught me the value of caring and supportive attitudes from medical professionals. I aim towards setting the right example for the medical students and residents whom I train.

## CONCLUSIONS

The physical and emotional hardships and trials following laryngectomy render the patient helpless and dependent. Receiving skillful, caring, and compassionate care is invaluable during that period.

Patient care can be improved by preparing the patients and their families for the medical, psychological, and social repercussions of the treatments received; by paying greater attention to the patients and spending more time with them; by keeping them updated and cognizant about their medical condition; by applying proper procedures in nursing care; by preventing medical mistakes through greater vigilance; and by training physicians and nurses how to respond appropriately to the unique post-surgical needs of their patients.

## Supplementary Information



## Abbreviations

SLP: speech and language pathologist
