# Supplementary material for: Cancer of the Throat: A Physician’s Experience as a Patient
Source: Rambam Maimonides Med J. 2016 Jul 28;7(3):e0025. doi: 10.5041/RMMJ.10252 (PMC5001797; doi:10.5041/RMMJ.10252)
Supplement: Supplementary file 1 [file rmmj-7-3-e0025-supplement.docx]

This appendix has been provided by the author to give readers additional information

Supplement to

Brook I. Cancer of the Throat: A Physician’s Experience as a Patient. Rambam Maimonides Med J 2016;7 (3):e0025. doi:10.5041/RMMJ.10252

# About the author

Dr Itzhak Brook is a Professor of Pediatrics at Georgetown University Washington DC. He graduated from Hareali Haivery High school in Haifa, earned his medical degree and completed his residency at Hebrew University, Hadassah School of Medicine, in Jerusalem, and obtained his master’s degree in pediatrics from the University of Tel Aviv in Israel. Subsequently he completed a fellowship in adult and pediatric infectious diseases at the University of California, Los Angeles. He served in the Medical Corps of the US Navy for 27 years.

Dr Brook is the past chairman of the Anti-infective Drug Advisory Committee of the Food and Drug Administration. He has done extensive research on anaerobic and respiratory tract infections, anthrax, and infections following exposure to ionizing radiation.

He is the author of six medical textbooks, 140 book chapters, and over 700 scientific publications. He is an editor, associate editor, and member of the editorial board of several medical journals and the Head and Neck Cancer Alliance.

Dr Brook was diagnosed with throat cancer in 2006 and underwent laryngectomy in 2008. He is the author and publisher of *My Voice: A Physician’s Personal Experience with Throat Cancer* (2009), *The Laryngectomee Guide* (2013), *In the Sands of Sinai: A Physician’s Account of the Yom Kippur War* (2011), and author of the Marcel Dekker-published volume *Pediatric Anaerobic Infections: Diagnosis and Management* (2002).

Dr Brook is the recipient of the 2012 J. Conley Medical Ethics lectureship by the American Academy of Otolaryngology–Head and Neck Surgery.
